# Interfacial gap formation of class II composite restorations with proximal box elevation using bulk-fill materials: a micro-CT study

**DOI:** 10.1038/s41598-025-30330-9

**Published:** 2025-12-04

**Authors:** Ceren Deger, Ayse Aslı Senol, Pınar Yılmaz Atalı, Burcu Oglakci Ozkoc

**Affiliations:** 1 Department of Restorative Dentistry, Faculty of Dentistry, Bezmialem Vakif University, Fatih, Istanbul, Türkiye; 2 Department of Restorative Dentistry, Faculty of Dentistry, Marmara University, Maltepe, Istanbul, Türkiye

**Keywords:** Bulk-fill composite, Proximal box elevation, Deep margin elevation, Micro-computed tomography, Gap formation, Health care, Materials science, Medical research

## Abstract

To evaluate the effect of proximal box elevation (PBE) using different bulk-fill resin composites on interfacial gap formation of Class II direct restorations at the cervical region using micro-computed tomography (micro-CT). Standardized two separate box-shaped Class II cavities were prepared on both mesial and distal surfaces of 25 sound human lower molars. Cavities were divided into five groups (n = 10) based on different bulk-fill composites used for PBE: (1) Group SB: sonic-activated bulk-fill, (2) Group EB: low-viscosity bulk-fill, (3) Group VB: thermo-viscous bulk-fill, (4) Group AB: bioactive dual-cure bulk-fill, and (5) Group FZ: no PBE (control)-conventional resin composite. A dual-cure universal adhesive was applied in self-etch mode. In PBE groups, a 3 mm bulk-fill layer was applied before layering conventional composite, while no PBE group was restored using conventional composite. Specimens underwent thermocycling (10,000 cycles, 5–55 °C). Micro-CT analysis measured interfacial gap formation based on radiolucent areas at the gingival floor of tooth-restoration interface. Data were analyzed using Welch ANOVA and Games-Howell post hoc tests (*p* < 0.05). Groups SB and VB showed significantly higher gap formation than Groups EB and AB, as well as Group FZ(control/no box elevation) (*p* < 0.05). No significant differences in gap formation were found between Groups EB, AB, and FZ (*p* > 0.05). PBE with low-viscosity and bioactive dual-cure bulk-fill composites effectively minimized interfacial gaps at the cervical region, suggesting their suitability for deep Class II restorations. PBE using low-viscosity bulk-fill composite resin and bioactive dual-cure bulk-fill composite resin could be preferred due to superior cavity adaptation in the cervical region compared to no proximal elevation.

## Introduction

The success of dental restorations is evaluated based on their achievement of clinical outcomes such as functionality and aesthetics. For restorative materials, the desired features include optimal mechanical and physical durability, as well as the ability to form ideal bonds with dental tissues and minimal technique sensitivity during application. However, resin composites, one of the most commonly used restorative materials, have varying clinical lifespans depending on the oral environment and the functional load. The formation of secondary caries, microleakage, and fractures are the most frequently observed failures that limit the clinical lifespan of composite restorations^1^. Microleakage in composite restorations may occur due to shrinkage stresses during polymerization, differences in the coefficient of thermal expansion between the restorative material and the tooth structure, and inadequate hybridization caused by fluid trapped in the gap between the adhesive system and collagen fibrils^2^.

In cavities extending below the cementoenamel junction, the presence of subgingival margins lacking enamel tissue creates significant technical challenges that may lead to microleakage^3^. Moreover, bonding to deep dentin tissue is more challenging than enamel tissue due to dentin tissue’s high organic content, tubular structure, and increased permeability^4^. In such cases, the biological width required between the alveolar crest and the restoration margin, as well as the conditions required for restoration procedures, can be achieved through surgical crown lengthening or orthodontic tooth extrusion^5^. However, in cases where invasive procedures cannot be performed, the subgingival margin can be repositioned supragingivally using resin composite material as a non-invasive alternative to surgical and orthodontic treatments. This technique is known as “cervical margin relocation” and has also been referred to over the years using various terms, such as “deep margin elevation” or “proximal box elevation”^3^.

Despite the advantages of proximal box elevation, the use of this technique remains a topic of debate due to the creation of an additional adhesive layer surface prone to leakage and the potential negative impact of these interfaces on the success of the restoration. The conflicting results from studies evaluating the feasibility of this technique highlight the importance of selecting the appropriate materials and techniques^3,6^.

Among the preferred resin composites for the proximal box elevation technique are bulk-fill resin composites, which can be placed in a single layer up to 4–5 mm thick and polymerized in one step^7^. As with conventional resin composites, there are also multiple types of bulk-fill composites with different properties, such as various organic matrix and inorganic filler contents. The viscosity of these composites also varies according to the application technique used^2^. Indeed, low-viscosity bulk-fill composites may be considered for use in deep margins to facilitate cavity application^8^. In a study, İsmail et al.^9^ evaluated the effect of various restorative materials—including high-viscosity glass ionomer, resin-modified glass ionomer, and active and flowable bulk-fill composites—on marginal and internal adaptation following proximal box elevation. Based on two-dimensional cavity adaptation analysis conducted using SEM, it was reported that flowable bulk-fill composite and bioactive materials exhibited superior cavity adaptation compared to resin-modified and high-viscosity glass ionomers. Similarly, Scotti et al.^10^ demonstrated that the use of flowable composites beneath different nanohybrid composites in deep margin elevation resulted in better cavity adaptation than non-flowable composites. This characteristic has encouraged research into alternative methods to temporarily reduce the viscosity of bulk-fill resin composites and improve adaptation without compromising the mechanical properties of the restoration. According to manufacturer information, innovative bulk-fill composite resins using thermo-viscous technology and sonic activation technology have been designed to allow for viscosity modulation^11^. The recently introduced thermoviscous bulk-fill composite is specifically formulated for preheating prior to clinical placement. As stated by the manufacturer^12^, the use of near-infrared (NIR) technology facilitates rapid and uniform heating, allowing for immediate application with improved thermal homogeneity. This method is purported to minimize the adverse effects of rapid cooling, thereby enhancing cavity adaptation^13^.

Another bulk-fill material, Activa Bioactive Restorative, is made of a resin-modified glass ionomer composed of diurethane monomers, modulated through the addition of methacrylate-based monomers and hydrogenated polybutadiene. As a result of the bioactive properties of these materials, it is claimed that they are able to minimize gap formation at the tooth-restoration interface^14^.

A continuous and durable adhesion between the restorative material and dentin tissue ensures optimal stress distribution and long-term restoration success^15^. Therefore, evaluating the cavity adaptation of restorative materials is important for predicting the long-term clinical performance of the restoration^2^.

The aim of the present study was to evaluate the effect of proximal box elevation using different bulk-fill resin composites on interfacial gap formation of Class II direct restorations at the cervical region using micro-computed tomography (micro-CT).

The null hypothesis of this study was that the type of bulk-fill composite materials for proximal box elevation would not influence the interfacial gap formation of Class II composite restorations at the cervical region.

## Materials and methods

This in vitro study was approved by the Bezmialem Vakif University Ethics Committee of Non-Invasive Studies (Process no: 15.11.2022-2022/343). All methods were carried out in accordance with relevant guidelines and regulations. Informed consent was obtained from all subjects and/or their legal guardians.

### Specimen size calculation

The sample size was calculated based on the estimated effect size between groups according to the literature^16^. It was determined that 8 specimens (cavities) in each group were required for an estimated medium effect size of *d* = 0.50, with 80% power and a 5% type 1 error rate.

### Specimen preparation and restorative procedures

Twenty-five sound human mandibular first molars of comparable size and morphology, extracted for periodontal or prosthetic reasons were collected. Teeth presenting cracks, carious lesions, restorations, or other structural defects were excluded following visual inspection under × 10 magnification using a stereomicroscope (SMZ 1000, Nikon, Japan). Immediately after extraction, residual soft tissue and periodontal debris were removed using an ultrasonic scaler (Woodpecker UDS-E LED, Guilin, China), and the teeth were cleaned with prophylactic paste and a soft brush under running water. The cleaned specimens were stored in 0.1% thymol solution at 4 °C until use. The specimens were randomly assigned to five groups (n = 5) according to the restorative protocol and type of bulk-fill resin composite used. On each tooth, two standardized box-shaped Class II cavities were prepared on the mesial and distal surfaces, resulting a total of 10 cavities per group (n = 10) and 50 restorations in total. In this study, the individual cavity rather than the tooth served as the experimental unit, consistent with previous micro-CT investigations of marginal gap formation and interfacial adaptation^10,16–18^. The cavities were designed with the following dimensions: 4 mm buccal-lingual width, 3 mm mesio-distal width, and a depth of 1 mm below the cementoenamel junction (CEJ). A periodontal probe was used to verify the cavity dimensions, and a coarse diamond bur (FC Diamond, G&Z Instrumente, Austria) with a high-speed handpiece (200,000 rpm) and under water cooling was used to prepare the cavities. The burs were replaced after each preparation. The buccal and lingual walls of cavities were shaped to be parallel to each other, and the cavity surface margins lacked a bevel. After cavity preparation, all specimens were examined under × 10 magnification using a stereomicroscope to confirm the absence of cracks, caries, or structural irregularities in enamel and dentin. Teeth exhibiting any visible defects or non-standardized cavity geometry were excluded and replaced to ensure standardized cavity configuration and uniform specimen integrity across all groups. All the preparations were completed by a single operator (A.A.S.). All the materials used in the study are shown in Table 1.

**Table 1:** The restorative materials used in the study, their compositions, and manufacturers.

Restorative material	Type	Organic content	Inorganic content
SonicFill 3, Kerr, Orange, CA, USA	Sonic-activated Bulk-fill	Bis-EMA Bis-GMA TEGDMA	Oxides, aluminum, barium glass, silica and YbF_3_ filler (up to 81.5 wt% / 65.9 vol%), inorganic fillers (up to 75 wt% / 55 vol%) with a particle size range of 40 nm–10 μm
Estelite Bulk fill, Tokuyama, Tokyo, Japan	Low viscosity bulk-fill	Bis-GMA Bis-MPEPP TEGDMA	(70 wt%/56 vol%) Supra-Nano Spherical filler (200 nm spherical SiO_2_-ZrO_2_) - Composite Filler (include 200 nm spherical SiO_2_-ZrO_2_
VisCalor Bulk, Voco, Cuxhaven, Germany	Thermoviscous bulk-fill	Bis-GMA Aliphatic dimethacrylate	83 wt%/68 vol% inorganic fillers -glass ceramic fillers (particle size 1 μm) innovatively functionalized siliciumdioxide nano particles (size 20–40 nm)
Activa Bioactive Restorative, Pulpdent, Watertown, MA, USA	Bioactive dual-cure bulk-fill	Blend of diurethane Methacrylates with modified polyacrylic acid (44.6%)	Amorphous silica (6.7%) Sodium fluoride (0.75%)
Filtek Z250, 3 M, ESPE, USA	Conventional resin composite	Bis-GMA UDMA Bis-EMA TEGDMA	77.6wt%, 60vol% zirconia/silica
Futurabond U, Voco GmHB, Cuxhaven, Germany	Dual-cure adhesive system	HEMA, Bis-GMA, HEDMA, acidic adhesive monomer, urethane dimethacrylate, catalyst, silica nanoparticles, ethanol, and water	

Bis-EMA, bisphenol-A-ethoxylated-glycidyl dimethacrylate; Bis-GMA, bisphenol A-glycidyl methacrylate; TEGDMA, Triethylene glycol dimethacrylate; Bis-MPEPP, Bisphenol-A ethoxylate Di methacrylate; UDMA, urethane dimethacrylate; HEDMA, 2-hydroxyethyl dimethacrylate; YbF_3_, Ytterbium(III) fluoride; SiO_2_, silicon oxide (silica); -ZrO_2_, zirconium oxide.

The teeth with two separate mesial and distal cavities were randomly divided into five groups according to the different bulk-fill composite resins used for proximal box elevation (n = 10):

*Group SB* Proximal box elevated with Sonic-activated bulk-fill (SonicFill 3, Kerr Corp, Orange, CA, USA).

*Group EB* Proximal box elevated with Low-viscosity bulk-fill (Estelite Bulk Fill, Tokuyama, Tokyo, Japan).

*Group VB* Proximal box elevated with Thermo-viscous bulk-fill (VisCalor Bulk, Voco, Cuxhaven, Germany).

*Group AB* Proximal box elevated with Bioactive dual-cure bulk-fill (Activa BioActive Restorative, PulpDent, USA).

*Group FZ* No proximal box elevation-Conventional resin composite (control group) (Filtek Z250, 3 M ESPE, USA).

After the cavity preparations were completed, a metal matrix system (SuperMat assorted kit, KerrCorp, Orange, CA, USA) was used for the application of the restorative materials in each tooth. A dual-cure universal adhesive (FuturaBond U, Voco, Cuxhaven, Germany) was applied to the cavities using a microbrush for 20 s, followed by air-thinning for 5 s in self-etch mode. The adhesive was polymerized with an LED light-curing unit (LCU) (Valo, Ultradent, South Jordan, UT, USA) (1000 mW/cm^2^) for 10 s.

In Groups SB, EB, and VB, bulk-fill composites were placed in a single 3 mm increment and light-cured for 20 s from the occlusal direction (1000 mW/cm^2^). The remaining 2 mm occlusal portion was restored with a conventional resin composite (Filtek Z250, 3 M ESPE, St. Paul, MN, USA) applied in 2 mm increments, each light-cured for 20 s. In Group FZ, the entire cavity was restored with Filtek Z250 using 2 mm incremental layers, each polymerized for 20 s.

The light intensity was controlled during the whole process using a radiometer (Demetron LED Radiometer, Kerr Corp.). All restorative materials were applied by a single operator (C.D.) in line with the manufacturer’s instructions.

Subsequently, the specimens were stored in distilled water at room temperature for 24 h. Afterward, all the samples underwent thermal aging in a thermal cycling device (SD Mechatronik Thermocycler THE-1100, Germany) set to 10,000 cycles between 5 and 55 °C.

### Micro-CT analysis

**Fig. 1:**
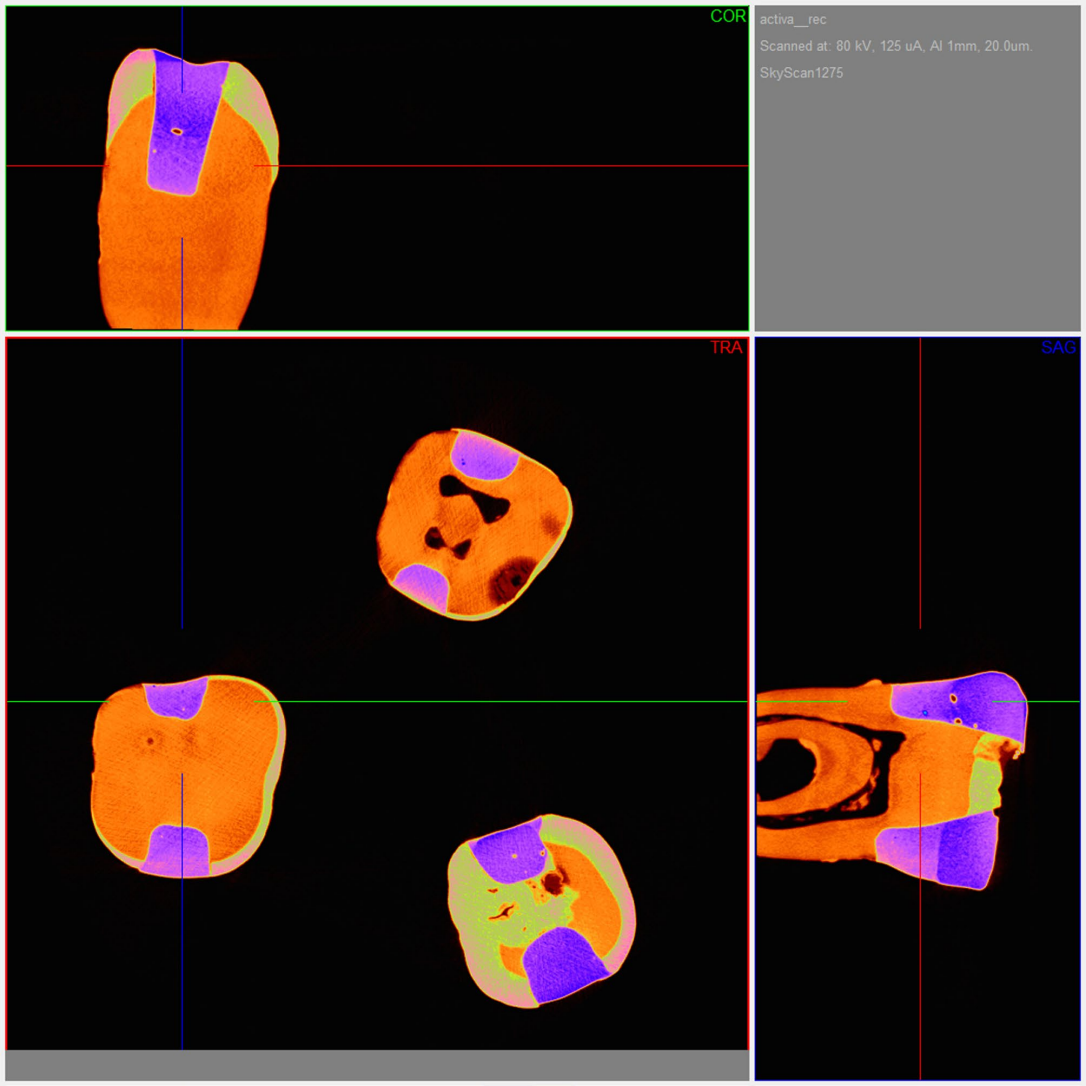
Representative two-dimensional cross-sectional image (ROI) with micro-CT NRecon software. Coronal, transverse, and sagittal sections reconstructed with NRecon software are shown to illustrate the standardized ROI used for gap analysis. The restored cavity (rendered in purple) and tooth structure (orange) are visualized in three planes to ensure consistent orientation and uniform assessment of the tooth-restoration interface.

**Fig. 2:**
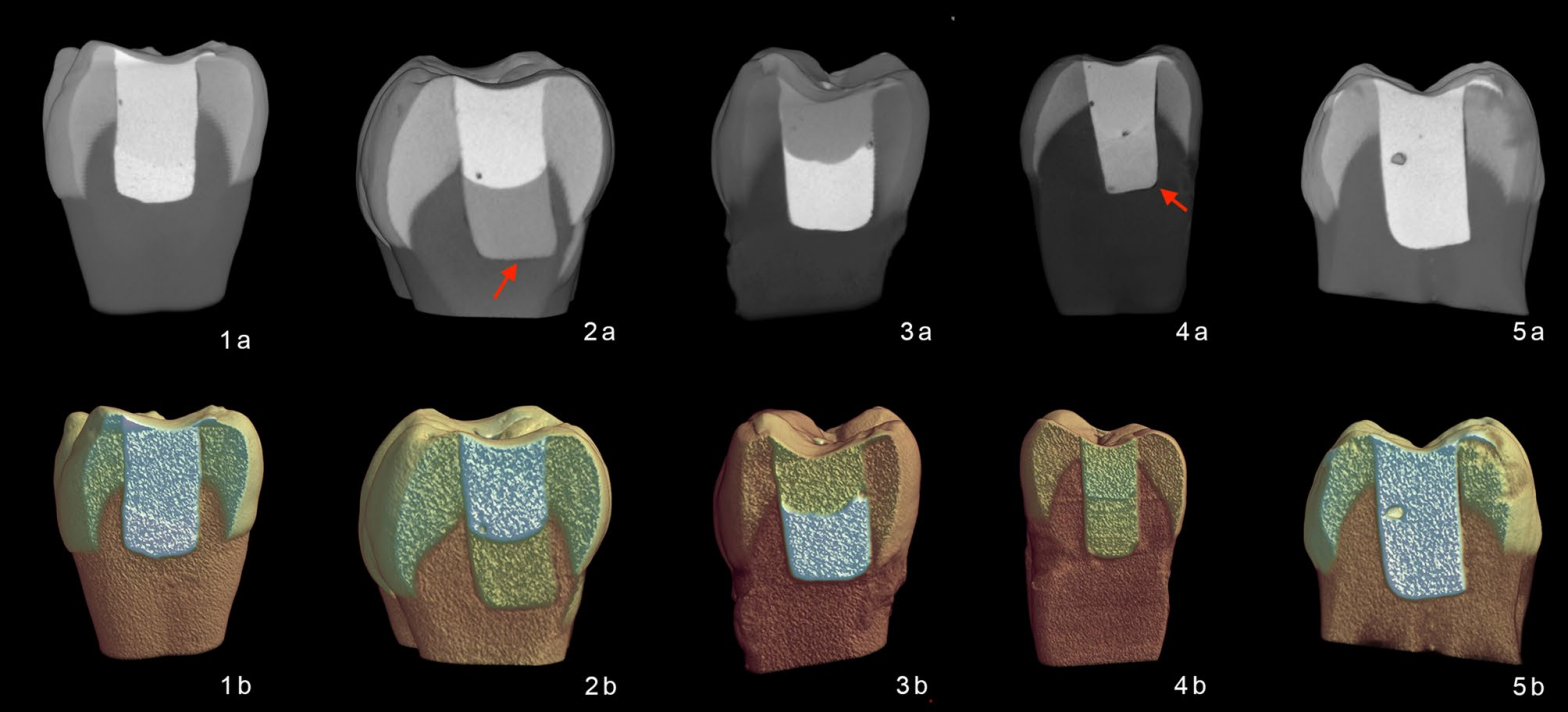
Two-dimensional and three-dimensional representative Micro-CT images of the samples (1a-1b: Group SB; 2a-2b: Group EB; 3a-3b: Group VB; 4a-4b: Group AB; 5a-5b: Group FZ). The upper row shows two-dimensional cross-sectional images illustrating the internal morphology of the restored cavities. Red arrows highlight radiolucent discontinuities indicating interfacial gaps, primarily located at the gingival floor. The lower row displays the corresponding three-dimensional segmented reconstructions.

After thermal aging, the samples were scanned using a micro-CT device (SkyScan 1275, Kontich, Belgium). The scans were conducted with a pixel size of 18 μm, a resolution of 1944 × 1944, an exposure time of 49 ms, 50 W power, and an Al filter. NRecon software (Ver 1.7.4.6, Micro Photonics Inc) and the CTAn system (Ver 1.19.11.1, SkyScan, Micro Photonics Inc) were used for the scanning and measurement of the samples (Fig. 1). The algorithm from Feldkamp et al. was employed for the reconstruction of two-dimensional axial slices. After scanning, the cavity adaptations of the restorations at the gingival step were evaluated. Radiolucent areas in the corresponding volumes of interest (VOI) created from the two-dimensional images of the region of interest (ROI) were detected (Fig. 2). The ROI was selected at the gingival floor of each cavity encompassing as standardized 4 × 3 mm-wide surface area. Approximately 339 transverse tomographic sections were obtained in bitmap file format (BMP). Radiolucent areas in this region of tooth-restoration interface were quantified in millimeters cubed (mm^3^) using the CT-Volume program (Ver 2.3.2.0, SkyScan, Micro Photonics Inc). The voids due to operator at the cavity line angles were excluded in the analysis. Two-dimensional and three-dimensional representative micro-CT images of the samples are shown in Fig. 2.

### Statistical analysis

The data were analyzed using IBM SPSS Statistics 22.0 software. The normality of the variables was assessed using the Shapiro–Wilk test, and the Levene’s test was employed to evaluate the homogeneity of variances. The data showed a normal distribution; however, the variances were not homogeneous. Differences between the multiple groups were assessed using Welch’s ANOVA. When the Welch’s ANOVA test was significant, pairwise comparisons were performed using the Games-Howell test (*p* < 0.05).

## Results

A comparison of the interfacial gap formation values at the tooth-restoration interface of the cervical region across the groups is presented in Table 2, with the values expressed in millimeters cubed (mm^3^). Significantly higher gap formation values were observed in Groups SB and VB compared to Groups EB and AB, as well as Group FZ (control/no box elevation) (*p* < 0.05). However, no significant differences in gap formation were found between Groups EB, AB, and FZ (*p* > 0.05).

**Table 2:** Mean interfacial gap formation values and standard deviations (± SD) (mm^3^) for all tested groups at cervical regions.

Groups	Mean ± SD
Group SB	0.268 ± 0.143^b^
Group EB	0.021 ± 0.017^a^
Group VB	0.423 ± 0.214^b^
Group AB	0.047 ± 0.037^a^
Group FZ (control/no elevation)	0.038 ± 0.033^a^
*p*	< 0.001

*Group SB: Proximal box elevated with Sonic-activated bulk-fill (SonicFill 3, Kerr Corp, Orange, CA, USA); Group EB: Proximal box elevated with Low-viscosity bulk-fill (Estelite Bulk Fill, Tokuyama, Tokyo, Japan); Group VB: Proximal box elevated with Thermo-viscous bulk-fill (VisCalorbulk, Voco, Cuxhaven, Germany; Group AB: Proximal box elevated with Bioactive dual-cure bulk-fill (Activa BioActive Restorative, PulpDent, USA); Group FZ: No proximal elevation/Conventional resin composite (control group) (Filtek Z250, 3 M ESPE, USA).

## Discussion

In this study, micro-CT was selected to assess the interfacial gap formation of box-shaped Class II composite restorations with proximal box elevation using different bulk-fill materials at the cervical region. Four types of bulk-fill composites (i.e., sonic-activated, low-viscosity, thermo-viscous, and bioactive dual-cure) used for proximal box elevation and one conventional composite were selected to treat proximal box-shaped Class II cavities. Our findings showed that teeth with proximal box elevations applied using sonic-activated bulk-fill and thermoviscous bulk-fill exhibited significantly higher gap formation compared to teeth in the other groups. Therefore, the null hypothesis that the type of bulk-fill composite materials for proximal box elevation would not influence the interfacial gap formation of Class II composite restorations at the cervical region, was rejected**.**

Various methods, such as air-pressure techniques, fluid infiltration, dye penetration, optical coherence tomography (OCT), and micro-CT, have been used to evaluate gap formation and microleakage at the tooth-restoration interface, with results from those methods showing significant differences^19,20^. Organic dyes such as basic fuchsine, methylene blue, and rhodamine are commonly used in conjunction with traditional laboratory techniques, such as microscopy or transmission electron microscopy, to detect microleakage. However, these methods have some limitations, such as being invasive, providing semi-quantitative results, and only representing the two-dimensional structure of the samples^21^. In contrast, micro-CT, a modern and non-destructive technology, allows for the generation of a three-dimensional visualization and quantitative data of the entire tooth structure without damaging the samples, making this technique the preferred analytical method in this study.

Although there is no clear correlation between gap formation observed in the laboratory and clinically observed interface failures, it is reasonable to assume that gap formation at the cervical region is clinically significant. Adhesion to dentin is influenced by various factors, including the morphological characteristics of the substrate, the type of adhesive system employed, and the precision of its application^22^. Numerous studies have demonstrated that achieving a complete marginal seal, particularly in deep cervical areas, remains difficult, thereby increasing the risk of microleakage^18,19^. Therefore, establishing a durable bond to deep cervical dentin and ensuring a long-term marginal seal of adhesive restorations remain inconsistent and clinically unpredictable. Clinicians commonly face challenges when restoring subgingival defects, considering the clinical conditions and the differing advantages of limitations of materials for restoration in this area.

The gingival margin of Class II restorations represents the most vulnerable area for recurrent caries and is commonly associated with maladjustments, misfits, and marginal gaps, particularly at the cervical region. Furthermore, when restorations extend below the cemento-enamel junction, the integrity of the marginal seal becomes questionable^23^. Han and Park^24^, who evaluated the cavity adaptation of bulk-fill composites in Class II cavities, reported that the highest level of imperfection was observed at the cervical region. The authors noted that the presence of enamel on the buccal and lingual walls of the cavities and its absence at the gingival margin contributed to adhesive failures in this region. Consistently, a previous study also showed that Class II cervical margins in dentin tissues exhibit greater marginal gaps compared to enamel tissues^25^. This finding is attributed to the hydrophilic nature of dentin, which makes it challenging for hydrophobic adhesives to penetrate effectively. Indeed, water may remain within the adhesive layer after solvent evaporation or migrate into the interface from the external environment, thus leading to a reduction in adhesion over time^26^. The long-term preservation of dentin bonding strength relies on maintaining a well-sealed interface.

Restorative materials are exposed to mechanical, thermal, and chemical stresses that induce fatigue damage, progressing from substructural alterations and microcrack initiation to structural degradation and eventual fracture^27^. Consequently, interfacial characterization is essential to clarify the mechanisms underlying biomechanical failure. To date, the majority of studies have focused on assessing the marginal adaptation of restorations relocated with composite resins in deep cervical regions. However, limited evidence is available regarding the interfacial adaptation of these restorations at cervical margins^28,29^. Therefore, the present study aimed to evaluate interfacial gap formation at the cervical region, where failures most frequently occur.

When evaluating the interfaces between resin composite and tooth structures, it should be considered that the adhesive system may affect the marginal and interfacial gap formation, particularly in cases where the margins are located apical to the CEJ. Due to the morphological and histological characteristics of dentin, the adhesion mechanism differs from that of enamel; indeed, dentin contains significant amounts of water and organic material, which can interfere with adhesion^30^. Therefore, considering also that light may not adequately reach the cavity floor in deep Class II cavities, thus potentially impairing polymerization, in this study, a dual-cure adhesive system was applied to all specimens in self-etch mode.

Cavity adaptation is influenced by various factors, including the viscosity and application method of the materials, their composition, polymerization shrinkage, and the stresses that may arise post-shrinkage^31^. In deep Class II restorations, achieving good adhesion requires the use of appropriate restorative materials and placement techniques. Conventional composite resins are placed in 2 mm increments to ensure proper cavity adaptation and polymerization; however, this approach is time-consuming and highly sensitive to the technique used^32^. A review reported that composite resin viscosity is not a determining factor for marginal quality, although applying the material in multiple thin layers is generally advisable^18^. Two studies demonstrated that the marginal adaptation of a restorative composite (Clearfil Majesty Posterior; Kuraray Noritake Dental Inc., Tokyo, Japan) to dentin was improved when applied in three consecutive 1-mm increments rather than in a single 3-mm increment for cervical margin relocation^33,34^. Moreover, several studies have recommended the use of highly filled flowable composites (e.g., Premise Flow; Kerr Corp.) or bulk-fill flowables (e.g., SureFil SDR Flow; Dentsply Pty. Ltd., Victoria, Australia) for proximal box elevation because of their favorable consistency and ease of handling^35,36^. Bulk-fill restorative materials incorporating modified initiator systems can further address polymerization-related challenges by ensuring adequate curing and reducing polymerization shrinkage stress, thereby improving cavity adaptation and minimizing interfacial defects, particularly along the gingival floor of cervical regions^2^. Additionally, the ability of bulk-fill composites to be placed in increments up to 4–5 mm simplifies clinical procedures and enhances efficiency^37^. In the present study, bulk-fill resin composites were applied in 3-mm increments for proximal box elevation, and the remaining portion of the restoration was completed with a conventional microhybrid composite. The microhybrid material was also used for the control group (without proximal box elevation) due to its well-established physicomechanical performance and recognition as the gold standard^38,39^.

Clinically, long-term adhesion requires the adhesive layer to resist hydrolytic degradation and the stresses caused by repeated thermal fluctuations. If the forces generated by polymerization shrinkage or thermo-mechanical stress exceed the bond strength, there will be an increased risk of gap formation at the restoration margins^40^. Therefore, in this study, 10,000 thermal cycles were applied to the restorations to simulate 1 year of clinical aging^41^.

Sonic-activated bulk-fill employs sonic energy to facilitate the application of the composite resin. It is claimed that when activated by sonic energy, the viscosity of this bulk-fill material decreases, thus imparting rheological properties that enhance cavity adaptation. In conditions with comparable polymerization shrinkage, it is known that restorative materials with lower viscosity provide better marginal adaptation. However, in our study, the micro-CT images revealed that the gap formation at the cervical region in the group with proximal box elevation applied with Sonic-activated bulk-fill was higher compared to in the other groups. Researchers have suggested that the reduction in viscosity of the composite resin due to the vibrations caused by sonic energy may lead to increased air entrapment and void formation. Furthermore, it has been proposed that sonication may coalesce small bubbles into larger voids, which could adversely affect the overall properties of this material^24,42^. In line with our findings, one study which evaluated the total gap formation of different bulk-fill composites in Class II MOD cavities with micro-CT, reported that earlier version of Sonic-activated bulk-fill caused more gaps compared to low viscosity bulk-fill.

Low viscosity bulk-fill has a lower filler content (70 wt%/56 vol%) due to its high ratio of organic matrix. Due to their lower viscosity, flowable composite resins are known to adapt better to cavity walls. Consequently, the reduced gap formation observed with low viscosity bulk-fill, comparable to the control group conventional microhybrid resin composite, can be attributed to its lower filler content and the spherical filler technology present in its inorganic matrix. It has also been reported the presence of larger gaps for some low-viscosity bulk-fill resins compared to conventional composites when using a light microscope for analyses.

A previous study reported that when microhybrid or nanohybrid composites are used for proximal box elevation, preheating is recommended to enhance handling and reduce the likelihood of interlayer gap formation^43^. Thermoviscous bulk-fill composites employ a preheating technique designed to decrease viscosity, enhance flowability, and improve adaptation to cavity walls, while preserving the material’s sculptability. Recent studies have suggested that thermoviscous bulk-fill shows fewer internal voids and better adaptation compared to conventional bulk-fill composites. However, in our study, there was greater gap formation with thermoviscous bulk-fill compared to the other tested composites. In one study, better marginal integrity was found for thermoviscous bulk-fill than for two other conventional bulk-fill composites in Class I cavities^38^. Similarly, it has been evaluated that the use of different insertion techniques (i.e., conventional, sonic, and preheating) using micro-CT in two separate studies and reported that preheating significantly improved the internal adaptation for all of the bulk-fill composites, including thermoviscous bulk-fill^44,45^. However, the authors reported that sonic insertion was effective in reducing voids only for Sonic-activated bulk-fill and nanofilled bulk-fill. However, these three studies did not apply any aging procedures to the specimens, whereas our study used thermal aging. Indeed, previous research^46^. indicates that aging negatively impacts marginal adaptation, which may explain the greater gap formation observed with Thermoviscous Bulk-fill in our study.

It has been suggested that bioactive restorative materials can promote the movement of ions at the adhesive interface, thereby increasing the mineral content and reducing nanoleakage^47^. Released fluoride ions may inhibit the activity of pro- and active MMP-2 and MMP-9, thus preventing adhesive degradation by facilitating the transfer of phosphate and calcium ions through the hybrid layer. These ions can form Ca-PO4/MMP complexes that inhibit MMP activity, precipitate, and crystallize^14^. In our study, the lower gap formation observed for the proximal box elevation group using Activa BioActive Restorative compared to the control group (no proximal box elevation) may be explained by this mechanism. Similarly, in a micro-CT study, different bioactive restorative materials and a conventional nanohybrid resin composite were examined in terms of their internal adaptation with dentin; notably, Activa BioActive Restorative was found to result in fewer gap formations compared to other groups, aligning with the results of our study.

^48^. One limitation of our study is the inability to adequately simulate the oral environment due to the storage of the teeth in distilled water instead of artificial saliva. This factor should be considered, as it may influence the long-term behavior of resin composites and the bioactivity potential of the bioactive dual-cure bulk-fill composite resin material. Another limitation of the present study was the lack of mechanical aging, which may have limited the detection of potential changes occurring under long-term functional loading. Repeated mechanical stresses over time can induce fatigue and weaken the adhesive interface. When localized stresses exceed the interfacial fracture toughness, crack initiation may occur, potentially resulting in additional gap formation^30^. Moreover, the relatively small number of specimens per group and the single post-aging scanning procedure should be acknowledged, as these constraints were influenced by the high cost of micro-CT analysis. In this study, restorations were scanned and analyzed following the aging process; however, future micro-CT investigations should incorporate multiple time-point analyses both before and after aging to better characterize the temporal progression of gap formation in restorative materials. Despite these limitations, the micro-CT technique remains a highly informative and non-destructive method for detailed three-dimensional evaluation of internal adaptation.

Although proximal box elevation enables the margins of the definitive indirect restoration to be positioned supragingivally, the composite-tooth interface created during the procedure often remains in a deep subgingival location. Margins placed in this region may still pose biological risks, including gingival inflammation, periodontal attachment loss, and localized bone resorption^49^. Therefore, robust clinical investigations are needed to clarify whether the proximal box elevation technique can reliably function as an alternative to surgical crown lengthening or orthodontic extrusion in situations where maintaining an appropriate biological width is difficult.

## Conclusion

Despite these limitations, when used for proximal box elevation in deep Class II cavities, low-viscosity bulk-fill composite resin and bioactive dual-cure bulk-fill composite resin demonstrated promising outcomes by exhibiting lower gap formation at the cervical region, than sonic-activated bulk-fill and thermoviscous bulk-fill. Moreover, proximal box elevation with those bulk-fill materials caused superior cavity adaptation in the same region to no proximal elevation.

## Data Availability

All data generated or analyzed during this study are included in this published article.
